# Testing the “Zero‐Sum Game” Hypothesis: An Examination of School Health Policies and Practices and Inequalities in Educational Outcomes

**DOI:** 10.1111/josh.12889

**Published:** 2020-03-03

**Authors:** Sara J. Long, Hannah Littlecott, Jemma Hawkins, Gemma Eccles, Adam Fletcher, Gillian Hewitt, Simon Murphy, Graham F. Moore

**Affiliations:** ^1^ DECIPHer, UKCRC Centre of Excellence, Cardiff University, 1‐3 Museum Place Cardiff CF10 3BD UK; ^2^ Department of Public Health, Environments and Society, London School of Hygiene & Tropical Medicine, 15‐17 Tavistock Place London WC1H 9SH UK; ^3^ DECIPHer, Cardiff University, 1‐3 Museum Place Cardiff CF10 3BD UK

**Keywords:** public health, organization and administration of school health programs, health improvement, health policy, inequalities, education outcomes attainment

## Abstract

**BACKGROUND:**

Health and education are intrinsically linked, while both are significantly patterned by socioeconomic status throughout the life course. Nevertheless, the impact of promoting health via schools on education is seen by some as a “zero‐sum game”; ie, focusing resources on health improvement activity distracts schools from their core business of educating pupils, potentially compromising educational attainment. There is emerging evidence that school health improvement interventions may beneficially influence both health and attainment. However, few studies have examined the relationship between school health improvement activity and socioeconomic inequalities in educational attainment.

**METHODS:**

Wales‐wide, school‐level survey data on school health policies and practices was linked with routinely collected data on academic attainment. Primary outcomes included attendance and academic attainment at age 14 (Key Stage 3) and 16 (Key Stage 4). Linear regression models were constructed separately for high and low Free School Meal (FSM) schools, adjusting for confounders. Interaction terms were fitted to test whether there was an interaction between FSM, health improvement activity, and outcomes.

**RESULTS:**

There were positive associations between almost all school health variables and KS3 attainment among high, but not low FSM schools. Similarly, for attendance, there were positive associations of several health variables among high but not low FSM schools. There were no associations for KS4 attainment.

**CONCLUSIONS:**

Our findings did not support the “zero‐sum game” hypothesis; in fact, among more deprived schools there was a tendency for better attendance and attainment at age 14 in schools with more embedded health improvement action.

Throughout the life‐course, health and education are intrinsically linked,^1^ with a synergistic interaction between the two.^2^ Better health during childhood impacts positively on educational attainment,^3^, ^4^, ^5^, ^6^, ^7^ whereas educational performance is associated with better health in adulthood.^8^, ^9^ Socioeconomic inequalities in health and education become entrenched during school years before carrying forward into adulthood.^10^, ^11^ In industrialized countries, schools provide access to most young peopleThus, schools are a key setting for universal interventions that can have population level impact while also impacting on socioeconomic inequalities.^12^ The World Health Organization's (WHO)^13^ Health Promoting Schools framework (HPS; WHO, 1997)^14^ advocates orientation of the whole school system toward health, resulting in a cultural shift toward the provision of a health enhancing environment. The HPS Framework identifies the following three areas through which schools can improve health: embedding health within the curriculum; promotion of health and well‐being through systemic changes to the school ethos/environment; and external engagement with families and the community. Consistent with Ottawa charter principles, the HPS framework places emphasis on changing the settings that individuals interact with, as opposed to changing the behavior of individuals within settings. There is evidence for the effectiveness of HPS approaches in improving outcomes such as physical activity, fitness, fruit and vegetable intake, smoking, and bullying.^15^, ^16^, ^17^

A common theme within the literature on a settings‐based approach to health improvement is that system change is more likely where there is demonstrable return‐on‐investment for the core business of the system;^18^ in the case of schools, academic attainment. Langford et al.^1^, ^16^ highlighted that few studies of school health improvement interventions measure effects on education attainment, and the educational impacts of such interventions are not well understood. This limited engagement with core business by school health researchers is likely a key driver of the often poor implementation of HPS approaches.^1^ Where health researchers do not recognize or engage with dynamics that are directed toward de‐prioritizing anything outside of “core business,” health initiatives may become washed out^19^ rather than assimilated into the everyday functioning of school systems.^1^ In contexts such as England, education policy has moved toward maximizing academic attainment at the expense of developing personal skills, health and well‐being,^16^ driven by an assumption that focusing resource on health takes resource away from the core business of schools, ie, educational progression. Thus, promoting health via schools is seen by some as a “zero‐sum game.”^16^ This assumption is contradicted by evidence from countries that place greater emphasis on overall student development, well‐being, and social and emotional development such as Finland, Sweden, and Australia,^20^ which also have better academic performance. Our recent findings from the School Health Research Network (SHRN) in Wales^21^ provide evidence that schools in which health and well‐being is highly embedded do not perform worse in terms of academic attainment. Indeed, there was some evidence that schools with a more comprehensive health focus performed better than those with less, after adjustment for socioeconomic confounding.

In education systems such as England and the United States, where schools narrowly target education attainment, it is not uncommon for schools to focus on students with better capabilities at the expense of less able students, which can result in increasing inequalities and disengagement of students from disadvantaged backgrounds.^2^ The result of teaching to the test, ie, to improve examination marks, may be harmful for the mental health of students.^22^ In contrast, in Wales and Scotland education systems are increasingly focusing on well‐being as one of the pillars of education (eg, the Donaldson review),^23^ with the aim that young people become well‐developed and educated so that they can contribute effectively to society. Further, consistent with the Ottawa Charters' notion of “healthy public policy”, also known as “health in all policies”,^24^, ^25^ the Wellbeing of Future Generations Act^26^ recognizes that all public bodies have a responsibility to ensure health and well‐being. This Act places significant emphasis not only on considering the population health implications of all policies, but also the creation of an equitable society.

The role of schools, and more broadly education systems, in mitigating or perpetuating socioeconomic inequality has been a subject of significant debate. Our recent analyses of data from Wales show that the majority of difference between schools in attainment outcomes can be explained by socioeconomic composition;^21^ indeed, it is naïve to think that schools can entirely compensate for society.^27^, ^28^ Research has only recently begun to investigate effects of schools on health inequalities, and although there is growing evidence that schools can help to reduce inequalities, it is possible that schools can have the opposite effect and make disparities in health outcomes between rich and poor children worse.^29^, ^30^, ^31^ One study^31^ examined the role of socioeconomic status (SES) and relationships at school. Findings suggested that social dynamics within schools, relating to young people's position within the school social hierarchy, may amplify or reduce inequalities. In schools which emphasized health and well‐being, there were narrower socioeconomic inequalities in pupils' subjective well‐being. In schools which prioritized pupils' well‐being, young people from poorer backgrounds may be more likely to feel connected to their school, which has implications for engagement with its instructional and regulatory orders,^32^ and in turn health, well‐being, and educational performance. However, to date, no studies have focused on the influence of health improvement interventions on educational inequalities. One previous review in this journal found positive effects on attainment of coordinated school health promotion programs targeting the curriculum and school environment.^5^ A literature review on the connection between health and attainment using the Whole School, Whole Community, and Whole Child (WSCC) framework reported that schools can improve health and learning by creating positive school health environments, as well as providing school health services, and engaging families and community.^33^ Impacts on socioeconomic inequality in education have received less attention. In the 2014 review of the WHO HPS framework^16^ just two of 67 studies reported effects on health outcomes by SES; none did so for academic attainment.

The primary aim of this study is to explore the role of embeddedness of health and well‐being in schools in reducing, or widening, socioeconomic inequality in educational attainment. The study also explores the notion that schools have nothing to gain, and may in fact experience detriments to academic outcomes as a result of focusing resources on health improvement activity. In light of evidence that there is a synergistic interaction between health and education, we hypothesize that to the contrary, there will be no evidence of a negative impact of health improvement on socioeconomic inequalities in educational attainment.

## METHODS

### Participants and Recruitment

This study used school‐level data collected from secondary schools in Wales that are members of the Wales‐wide SHRN.^34^ The School Environment Questionnaire (SEQ), collected in 2016 was completed by school staff. The SEQ includes questions on organizational structures for delivery of health improvement, and the presence, breadth, and depth of school health improvement activities. At the time of the survey, network schools represented just over half of all secondary schools in Wales (N = 115/212; 53%), with representation in all 22 local authority areas. Schools were recruited to the network through three mechanisms. Firstly, those participating in the Welsh Health Behaviour in School‐aged Children (HBSC) survey in 2013/2014^35^ were invited to join (60 out of 82 secondary schools approached joined the network). Secondly, nine schools in South Wales that were recruited to a HBSC substudy in 2013 to pilot data linkage methods joined the network. Finally, 44 schools joined in 2015 during a period of open recruitment. Each member school had a designated member of staff who acted as a contact person and they were briefed about the survey via emails, newsletters, and at an event for schools in June 2015.

### Instrumentation

#### *Socioeconomic status*

Routinely available data on the percentage of students entitled to Free School Meals (FSM), within each school were obtained from http://mylocalschool.wales.gov.uk and used as an indicator of the socioeconomic composition of the school. Recent studies have demonstrated the validity of this measure as an indicator of school‐level deprivation.^36^ The national median percentage was used to define schools as having either low or high FSM entitlement. Schools with less than 15% of students entitled to FSM (ie, the median value) were defined as “low FSM” and schools with more than 15% of students entitled to FSM were defined as “high FSM.”

#### *Educational attainment*

Routinely available school level data were obtained from http://mylocalschool.wales.gov.uk. Outcomes included attendance (%) and pupils within each school who reached the expected level in core subjects (%; English or Welsh, Maths, and Science) by the end of Key Stage 3 (KS3; 11–14 years) and attaining qualifications equal to 5 general certificate of secondary education at A–C grade, including English/Welsh and mathematics at Key Stage 4 (KS4; 14–16 years). http://mylocalschool.wales.gov.uk

#### *Physical and mental health in school curriculum*

Schools were presented with a grid for each of a range of topic areas (physical activity, diet, drugs, tobacco, alcohol, sex education, and mental health) and asked to indicate which year groups received health education in that topic, and in which subject areas (PSE or Welsh Baccalaureate, Science, Vocational courses, Other, not taught to this year group). For each item, a sum score was generated, providing an overall indicator of the embeddedness of health into the curriculum. Sum scores were then subjected to factor analysis. Items relating to physical health (physical activity, diet, drugs, tobacco, alcohol, and sex education) demonstrated loadings greater than 0.4 on the first factor and formed a scale with a Cronbach's alpha coefficient of 0.83, indicating good internal consistency. The single item on mental health education within the curriculum was the only item not to load onto this factor. Hence, two variables were constructed: (1) physical health education in the curriculum and (2) mental health in the curriculum.

#### *School health policies*

Schools were asked to indicate which of a list of health and well‐being areas were covered by a written policy within their school. These were food and fitness, smoking, drugs, alcohol, mental health, suicide prevention, and violence against women and girls. A sum score was constructed to indicate the number of areas covered by schools' written policies.

#### *Involvement of students in developing health improvement policies*

Schools were asked about the extent to which students were involved in developing policies on health and well‐being, smoking and tobacco use (including drugs), healthy eating or food and fitness, mental health and well‐being, behavior and discipline, bullying, suicide prevention and/or post suicide care, sex and relationships, and violence against women and girls. Responses ranged from no student involvement, consultation with school council, consultation with other student voice groups, wider consultation with student suggestion boxes, and other. A variable was created by summing the number of policy areas (ie, no student involvement versus any student involvement) in which schools reportedly involved students.

#### *Involvement of parents in health improvement*

Schools were asked to estimate what proportion of parents were involved in health improvement activities in their school, with four options ranging from none to all. Schools were also asked in what areas parents were involved in health improvement (deciding on health priority areas, delivery of health education, development of school health policies, and other), and what mechanisms were used to involve parents (parent‐teacher association meetings, parent information evenings, parental surveys, involvement initiated by parents, through parent governors, and in one‐to‐one meetings). Three items were derived and subjected to factor analysis: the proportion of parents involved in health improvement; the number of areas in which parents were involved; and the number of mechanisms for involving parents. All items loaded onto a single factor (>0.6), and formed a scale with an alpha coefficient of 0.66, indicating acceptable internal consistency.

#### *Partnerships*

Schools were asked to indicate any formal or informal partnerships beyond statutory requirements to help students remain or become physically active (eg, with families, other schools, local community groups, professional sports clubs, Sports Wales or other national sport bodies, private sector businesses or organizations, local authority sports development officers, the local health board, or other). The presence of a partnership was given a score of 1, thus overall scores ranged from 0 to 9. Partnerships were used as a proxy for how well networked schools were.

#### *Organizational commitment to health*

Schools were asked to select up to 4 areas which were prioritized by senior management in the past 2 academic years from a list of 10 areas, including student emotional and mental health, student physical health, staff health and items on educational performance, and school environment. A score of 0 was assigned if no student health item was selected, “1” if one was, and “2” if both were. Schools were also asked if they had a written action plan for student health, and how often this was reviewed. A score of 0 was assigned if there was no action plan, 1 for action plans that were reviewed less than once a year and 2 if there was an action plan reviewed annually. These items were summed to form an ordinal scale scored from 0 (lowest level of organizational commitment to health) to 4 (highest level of organizational commitment to health).^37^

#### *Overall embeddedness of health in the school health curriculum*

A composite measure of embeddedness of health improvement was derived through grouping items into the three domains of the WHO's HPSs framework (ie, curriculum, environment/ethos, and parental/community engagement). Curriculum was the sum of items for physical and mental health education in the curriculum. School environment/ethos was the sum of items including written health policies and student involvement in health improvement. Parental/community engagement was the sum of items for parental involvement in health improvement and partnership working. Scores for each domain were scaled from 0 to 1 before summing, such that a score of 0 indicated lowest possible embeddedness of health improvement, and 3 the highest possible embeddedness.

### Data Analysis

First, Spearman's rank correlation coefficients were used to examine unadjusted associations between all variables. Subsequently, to test whether health improvement activity was associated with inequality in attendance and attainment, linear regression models were constructed separately for high and low FSM schools for each of the health improvement variables, adjusting for FSM entitlement 2016, and KS3 attainment and attendance in 2013. Finally, interaction terms were fitted to test whether there was an interaction between FSM and health improvement activity in predicting attendance and attainment.

## RESULTS

### Sample Characteristics

Out of 115 member schools, a response was received from 100 schools(a response rate of 87%, representing 45% of all secondary schools in Wales). Three independent schools were excluded from analyses due to absence of standardized data on educational attainment. Participating schools were representative of all state maintained secondary schools in Wales in terms of FSM entitlement (mean = 16.9%; SD = 9.2), school size (mean = 907.4; SD = 356.8) and the percentage of young people achieving the expected level at KS3 (mean = 88.1; SD = 6.5). The final sample consisted of 48 low FSM and 49 high FSM schools.

### Bivariate Associations

Tables 1 and 2 show unadjusted associations between health improvement variables, attendance and attainment for low and high FSM schools, respectively. Among high FSM schools, attainment at KS3 was positively associated with having written health policies, higher parental and student involvement with health improvement activities, and greater overall embeddedness of health improvement. These associations were not observed for low FSM schools, nor KS4 attainment in high or low FSM schools. In high, but not low FSM schools, there was a positive association between attendance and organizational commitment to health, student involvement and partnerships.

**Table 1:** Unadjusted Associations Between Variables Among Low Free School Meal Schools

	Free School Meal Entitlement	Organizational Commitment	Physical Health in Curriculum	Mental Health Education	Written Policies	Parental Involvement	Student Involvement	Partnerships	Overall Embeddedness of Health	Key Stage 32013	Key Stage 42013	Attendance 2013	Key Stage 32016
Organizational commitment	−0.31												
Physical health in curriculum	−0.10	0.19											
Mental health in curriculum	−0.01	0.08	**0.42**										
Written policies	**−0.53**	0.14	**0.34**	0.14									
Parental involvement	0.13	**0.37**	0.27	**0.29**	−0.14								
Student involvement	−0.01	**0.33**	0.15	0.13	−0.01	**0.47**							
Partnerships	−0.23	**0.28**	**0.29**	0.26	0.05	**0.27**	0.23						
Overall embeddedness of health	−0.16	**0.43**	**0.65**	**0.67**	**0.28**	**0.61**	**0.59**	**0.57**					
Key Stage 3 2013	**−0.51**	0.22	0.02	−0.16	0.06	−0.19	0.07	−0.09	−0.06				
Key Stage 4 2013	**−0.58**	**0.29**	0.12	−0.17	**0.33**	−0.17	0.21	0.11	0.16	**0.41**			
Attendance 2013	**−**0.27	0.14	−0.09	0.20	−0.02	0.09	−0.01	−0.05	0.03	0.17	0.11		
Key Stage 3 2016	**−0.28**	−0.02	0.05	0.09	0.24	0.06	0.01	0.14	0.09	0.12	−0.01	0.27	
Key Stage 4 2016	**−0.40**	0.07	0.24	0.09	0.22	0.10	0.21	0.02	0.21	0.19	0.36	0.12	0.22
Attendance 2016	**−0.43**	−0.09	−0.17	0.00	0.18	0.02	−0.07	−0.06	−0.08	0.16	**0.07**	**0.33**	**0.53**

Significant values are indicated in bold.

**Table 2:** Unadjusted Associations Between Variables Among High Free School Meal Schools

	Free School Meal entitlement	Organizational Commitment	Physical Health in Curriculum	Mental Health Education	Written Policies	Parental Involvement	Student Involvement	Partnerships	Overall Embeddedness of Health	Key Stage 32013	Key Stage 42013	Attendance 2013	Key Stage 32016
Organizational commitment	0.11												
Physical health in curriculum	**0.31**	−0.03											
Mental health in curriculum	0.14	0.24	0.07										
Written policies	0.07	**0.41**	0.14	0.15									
Parental involvement	0.10	**0.38**	0.19	0.19	**0.31**								
Student involvement	−0.11	**0.33**	0.27	**0.33**	0.08	**0.35**							
Partnerships	0.23	**0.38**	0.13	0.20	**0.27**	**0.43**	**0.28**						
Overall embeddedness of health	0.12	**0.50**	**0.35**	**0.56**	**0.55**	**0.73**	**0.62**	**0.59**					
Key Stage 3 2013	**−0.60**	0.05	−0.24	−0.21	−0.03	−0.10	0.06	**−0.30**	−0.17				
Key Stage 4 2013	**−0.64**	−0.03	−0.27	−0.06	−0.19	−0.12	0.19	**−0.39**	−0.19	**0.61**			
Attendance 2013	**−0.44**	−0.03	−0.13	−0.06	0.22	−0.21	−0.11	**−0.44**	−0.13	**0.63**	**0.42**		
Key Stage 3 2016	**−0.47**	0.25	0.18	0.06	**0.29**	**0.38**	**0.43**	0.01	**0.40**	**0.51**	**0.37**	**0.40**	
Key Stage 4 2016	**−0.44**	0.07	0.07	0.05	−0.09	0.13	0.22	−0.20	0.12	**0.45**	**0.49**	**0.34**	**0.51**
Attendance 2016	**−0.48**	**0.32**	0.00	0.22	0.13	0.18	**0.29**	**−0.28**	0.21	**0.56**	**0.50**	**0.60**	**0.64**

Significant values are indicated in bold.

### Multivariate Analyses

The results of the regression analyses are presented in Table 3, which includes coefficients from separate models for high and low SES schools, plus the interaction term from models including all schools (N = 97). For KS3 attainment, significant interactions were observed between FSM entitlement and embeddedness of physical health in the curriculum, parental and student involvement, overall embeddedness of health and organizational commitment to health. Main effects models showed that with the exception of mental health in the curriculum, there were positive associations between all school health improvement variables and KS3 attainment among high FSM schools only. There were no significant associations or interactions between FSM and health improvement variables for KS4 attainment. Significant interactions were observed between FSM and attendance for mental health in the curriculum, parental involvement, student involvement, overall embeddedness of health, and organizational commitment to health. These interactions reflected main effects of health improvement activities among high but not low FSM schools, with the exception of organizational commitment to health whereby there was a significant negative association with attendance in low FSM schools and a positive association in high FSM schools.

**Table 3:** Associations and Interactions Between Key Stage 3 (KS3) and Key Stage 4 (KS4) Attainment and Attendance in 2016, Free School Meal (FSM) Status, and Health Improvement Activities

	Co‐efficient (95% confidence intervals)					
	Sample N = 48	Sample N = 49	Sample N = 48	Sample N = 49	Sample N = 48	Sample N = 49
	KS3 Attainment 2016: Low FSM Schools	KS3 Attainment 2016: High FSM Schools	KS4 Attainment 2016: Low FSM Schools	KS4 Attainment 2016: High FSM Schools	Attendance 2016: Low FSM Schools	Attendance 2016: High FSM Schools
Regression analyses and interaction term for including physical health in the curriculum						
Attainment/attendance 2013	−0.05 (−0.15, 0.06)	0.13 (−0.03, 0.29)	0.20 (−0.08, 0.48)	**0.45 (0.05, 0.84)**	**0.25 (0.05, 0.44)**	**0.44 (0.24, 0.64)**
FSM 2016	**−0.39 (−0.72, −0.06)**	**−0.51 (−0.71, −0.30)**	−0.63 (−1.39, 0.14)	**−0.46 (−0.93, 0.00)**	**−0.08 (−0.14, −0.02)**	**−0.05 (−0.08, −0.01)**
Physical health in curriculum	0.02 (−0.04, 0.09)	**0.19 (0.07, 0.31)**	0.06 (−0.07, 0.18)	0.20 (−0.03, 0.44)	−0.01 (−0.02, 0.00)	0.01 (−0.01, 0.03)
Interaction	**0.01 (0.00, 0.02)**		0.01 (−0.01, 0.02)		**0.00 (0.00, 0.00)**	
Regression analyses and interaction term for including mental health in the curriculum						
Attainment/attendance 2013	−0.03 (−0.13, 0.08)	0.13 (−0.05, 0.30)	0.22 (−0.07, 0.51)	**0.42 (0.01, 0.84)**	**0.31 (0.10, 0.51)**	**0.42 (0.23, 0.62)**
FSM 2016	**−0.38 (−0.71, −0.05)**	**−0.44 (−0.66, −0.22)**	−0.60 (−1.38, 0.19)	−0.41 (−0.88, 0.07)	**−0.07 (−0.13, −0.01)**	**−0.05 (−0.08, −0.01)**
Mental health in curriculum	0.17 (−0.21, 0.56)	0.34 (−0.25, 0.94)	0.14 (−0.58, 0.86)	0.38 (−0.74, 1.50)	−0.04 (−0.10, 0.03)	**0.10 (0.01, 0.20)**
Interaction	0.03 (−0.01, 0.07)		0.03 (−0.05, 0.10)		**0.01 (0.00, 0.02)**	
Regression analyses and interaction term for having written health policies						
Attainment/attendance 2013	−0.05 (−0.15, 0.06)	0.15 (−0.02, 0.31)	0.21 (−0.07, 0.49)	**0.48 (0.07, 0.89)**	**0.27 (0.07, 0.47)**	**0.45 (0.24, 0.66)**
FSM 2016	−0.33 (−0.72, 0.06)	**−0.43 (−0.63, −0.23)**	−0.69 (−1.57, 0.19)	−0.36 (−0.82, 0.10)	**−0.09 (−0.17, −0.02)**	**−0.04 (−0.08, −4.78)**
Written policies	0.25 (−0.50, 1.00)	**1.14 (0.33, 1.95)**	−0.23 (−1.68, 1.22)	0.70 (−0.92, 2.31)	−0.06 (−0.20, 0.08)	0.01 (−0.14, 0.16)
Interaction	0.04 (−0.03, 0.10)		−0.00 (−0.13, 0.13)		−0.00 (−0.01, 0.01)	
Regression analyses and interaction term for having parental involvement						
Attainment/attendance 2013	−0.04 (−0.14, 0.06)	0.12 (−0.04, 0.28)	0.22 (−0.06, 0.50)	**0.46 (0.06, 0.86)**	**0.28 (0.08, 0.48)**	**0.48 (0.29, 0.66)**
FSM 2016	**−0.41 (−0.74, −0.08)**	**−0.46 (−0.65, −0.26)**	−0.63 (−1.40, 0.14)	−0.38 (−0.84, 0.08)	**−0.07 (−0.14, −0.01)**	**−0.04 (−0.07, −0.00)**
Parental involvement	0.22 (−0.10, 0.54)	**0.72 (0.30, 1.13)**	0.22 (−0.40, 0.84)	0.54 (−0.30, 1.39)	0.01 (−0.05, 0.07)	**0.10 (0.03, 0.17)**
Interaction	**0.04 (0.01, 0.07)**		0.03 (−0.03, 0.10)		**0.01 (0.00, 0.01)**	
Regression analyses and interaction term for having student involvement						
Attainment/attendance 2013	−0.04 (−0.14, 0.06)	0.11 (−0.05, 0.26)	0.17 (−0.12, 0.46)	**0.42 (0.01, 0.83)**	**0.28 (0.08, 0.48)**	**0.48 (0.30, 0.66)**
FSM 2016	**−0.40 (−0.73, −0.06)**	**−0.39 (−0.59, −0.20)**	−0.68 (−1.45, 0.10)	−0.37 (−0.83, 0.09)	**−0.07 (−0.14, −0.01)**	−0.03 (−0.06, 0.01)
Student involvement	0.02 (−0.36, 0.40)	**0.92 (0.42, 1.42)**	0.38 (−0.38, 1.14)	0.49 (−0.57, 1.54)	−0.01 (−0.09, 0.06)	**0.14 (0.06, 0.22)**
Interaction	**0.05 (0.02, 0.08)**		0.03 (−0.03, 0.09)		**0.01 (0.00, 0.01)**	
Regression analyses and interaction term for having partnerships						
Attainment/attendance 2013	−0.03 (−0.13, 0.08)	0.13 (−0.04, 0.30)	0.21 (−0.07, 0.49)	**0.46 (0.04, 0.88)**	**0.28 (0.08, 0.48)**	**0.46 (0.26, 0.67)**
FSM 2016	**−0.35 (−0.70, 0.00)**	**−0.48 (−0.69, −0.26)**	−0.67 (−1.46, 0.12)	−0.37 (−0.84, 0.09)	**−0.08 (−0.14, −0.01)**	**−0.04 (−0.08, −0.00)**
Partnerships	0.28 (−0.40, 0.95)	**0.83 (0.04, 1.63)**	−0.38 (−1.61, 0.85)	0.14 (−1.43, 1.71)	−0.03 (−0.15, 0.09)	0.03 (−0.11, 0.16)
Interaction	0.04 (−0.02, 0.10)		0.04 (−0.07, 0.14)		0.00 (−0.00, 0.01)	
Regression analyses and interaction term for overall embeddedness of health in the school						
Attainment/attendance 2013	−0.03 (−0.13, 0.07)	0.13 (−0.01, 0.27)	0.21 (−0.07, 0.49)	**0.45 (0.05, 0.85)**	**0.28 (0.08, 0.48)**	**0.44 (0.26, 0.62)**
FSM 2016	**−0.34 (−0.68, −0.01)**	**−0.49 (−0.67, −0.31)**	−0.58 (−1.36, 0.20)	−0.41 (−0.87, 0.04)	**−0.08 (−0.15, −0.02)**	**−0.05 (−0.08, −0.01)**
Overall embeddedness	1.49 (−0.87, 3.84)	**6.86 (4.19, 9.53)**	1.57 (−2.96, 6.10)	4.52 (−1.52, 10.54)	−0.26 (−0.70, 0.18)	**0.74 (0.24, 1.23)**
Interaction	**0.28 (0.09, 0.47)**		0.18 (−0.22, 0.57)		**0.05 (0.02, 0.09)**	
Regression analyses and interaction term for organizational commitment to health						
Attainment/attendance 2013	−0.04 (−0.14, 0.06)	0.10 (−0.07, 0.27)	0.23 (−0.05, 0.51)	**0.43 (0.02, 0.84)**	**0.29 (0.10, 0.48)**	**0.44 (0.26, 0.62)**
FSM 2016	**−0.45 (−0.80, −0.11)**	**−0.46 (−0.67, −0.25)**	−0.70 (−1.48, 0.08)	−0.39 (−0.86, 0.08)	**−0.09 (−0.16, −0.03)**	**−0.04 (−0.08, −0.01)**
Organizational commitment	−0.41 (−1.15, 0.32)	**1.41 (0.21, 2.61)**	−0.76 (−2.19, 0.67)	0.76 (−1.56, 3.08)	**−0.15 (−0.28, −0.02)**	**0.30 (0.12, 0.48)**
Interaction	**0.10 (0.02, 0.17)**		0.06 (−0.08, 0.20)		**0.02 (0.01, 0.03)**	

Significant values are indicated in bold.

## DISCUSSION

### Present Findings and Previous Literature

To our knowledge, this is the first study to examine the association of health improvement activity with socioeconomic inequality in educational attainment. Our results provide evidence of better attendance and attainment at KS3 in schools with more health improvement activity. Health improvement action may target a range of causal pathways that influence health and attainment and reduce inequalities.^32^, ^38^ For example, emphasis on staff‐student relationships may mitigate the association between disadvantage and poor outcomes.^38^ Studies from education demonstrate a greater emphasis on the provision of pastoral care and emotionally supportive relationships in poorer schools,^39^ and this may be important in supporting pupils' well‐being and engaging students from poorer backgrounds in learning. Notably, a lack of an observed association at KS4 may reflect a number of factors, eg, lower influence of schools and parents, and higher influence of peers, among this age group.^40^ Experimental evidence suggests that changes to the school environment to improve relationships between students and staff, and interventions that aim to reduce disengagement can lead to improvements in health behavior.^31^, ^41^, ^42^, ^43^, ^44^, ^45^ Whether this translates into improvements in education attainment requires further investigation.

Interventions that include environmental change components have been found to be more cost effective^46^ and less likely to create inequalities in health than purely educational interventions.^47^, ^48^ Our findings suggest that a greater embeddedness of health improvement has more beneficial outcomes among more deprived schools. While interventions that include family involvement can impact positively on student attainment,^49^, ^50^, ^51^, ^52^ family engagement is highly socially patterned, and family and community engagement components are often poorly delivered within “whole‐school” interventions.^1^ Our findings indicate that better parental engagement may be a potential mechanism through which inequalities can be reduced. The importance of student involvement has been noted,^41^, ^53^ with the extent to which students feel involved in the school decision‐making process, and staff‐student relationship quality, associated with better health outcomes.^15^, ^33^, ^42^, ^54^, ^55^ Our findings suggest that emphasizing student involvement in decision making may be particularly important for improving attainment in more deprived schools.

### Limitations

The study included a large, nationally representative sample of secondary schools in Wales. Whereas adjustment for earlier differences in attainment outcomes helps to reduce the risk of reverse causality, confounding cannot be ruled out. The reliance on self‐reported school health improvement activity by senior management team members provides a further limitation, as does the focus on quantity, rather than quality or evidence‐based nature of school‐level health improvement interventions. Although the SEQ was not designed to specifically capture the HPS framework, the measures map on to the three dimensions of the framework and provide an indication of the level of activities that can serve as proxy indicators.

The ecological nature of the data means that we are unable to rule out the ecological fallacy, and draw conclusions about what works for deprived pupils, as opposed to within deprived schools. Notably, we plan to include unique identifiers in future rounds of the Student Health and Wellbeing Survey, which is collected alongside the SEQ. This will enable longitudinal analyses of individual level health and education outcomes, and extension of these analyses to consider impacts of health improvement action on inequalities within, rather than just between, schools.

Finally, there is a significant need for evaluations of interventions to routinely incorporate analyses of effects on inequalities in health and attainment to establish what intervention characteristics should be targeted. Researchers examining the impact of interventions on inequalities need to move beyond outlining the epidemiology of such problems, to collaborate with policy and practice partners to begin to articulate scenarios for reducing inequalities. This requires working alongside communities, and as such a focus on parental and community involvement, inclusive of less affluent parents and communities.

### Conclusions

The present cross‐sectional study does not support the “zero‐sum game hypothesis,” and in fact, provides evidence to the contrary for KS3 attainment and attendance. The suggests that health improvement activity may be associated with better educational outcomes, particularly among low‐income schools.

## IMPLICATIONS FOR SCHOOL HEALTH

The WHO's HPS framework and the WSCC framework recommend several areas in which schools can improve health and learning simultaneously, including (1) making changes to the school climate (ie, environment and ethos); and (2) engaging with families and communities. In particular, environmental change interventions have been found to be the most effective components of health improvement activity within schools.

Consistent with the HPS and WSCC frameworks and existing evidence, the present study suggests effective health improvement activities to improve attendance and attainment at age 14 among low‐income schools are:

- Physical health in the curriculum. Where possible, this should be delivered in a variety of formats and subject areas, as opposed to delivery solely in separate health education classes.
- School health policies across a range of topics including food and fitness, smoking, drugs, alcohol, mental health, suicide prevention, and violence against women and girls.
- Student involvement in developing health improvement policies, where possible via a range of mechanisms (eg, consultation with the school council, consultation with other student voice groups, wider consultation with student suggestion boxes, student surveys).
- Involvement of parents in health improvement across a variety of areas. This may include deciding on health priorities, the delivery of health education, and/or the development of school health policies. Parents should be included via a range of mechanisms such as parent and teacher meetings, parent information evenings, parental surveys, and one‐to‐one meetings. A strategy for engaging with families from deprived backgrounds includes offering practical support for engagement (such as transport where no other means of attending the school is available); another is to communicate aspects of school policy that may help extend the school ethos and core values to the home.
- Informal and formal partnerships. These should be beyond statutory requirements, and should help students become or remain active. Partnerships may be with families, other schools, community groups, professional sports clubs, private sector organizations, and local government bodies.
- Prioritization of, and commitment to, key health and well‐being issues by senior management, including the production of written action plans.

Support to improve school health and education outcomes and reduce inequalities can be garnered from the wider school community by sharing the findings of this research with key stakeholders, including members of parent‐teacher associations or other organizations in order to highlight the importance of a commitment to health and well‐being as a means to address education inequalities.

### Human Subjects Approval Statement

Ethical approval for the survey was granted by Cardiff University School of Social Sciences Research Ethics Committee. The introduction to the SEQ described its purpose and how data would be used. Completion was taken as consent.

### Conflict of Interest

The authors declare no conflict of interest.
